# The roles of preventive and curative health care in economic development

**DOI:** 10.1371/journal.pone.0206808

**Published:** 2018-11-07

**Authors:** Fuhmei Wang

**Affiliations:** Department of Economics and Department of Public Health, National Cheng Kung University, Tainan, Taiwan; National Institute of Health, ITALY

## Abstract

**Introduction:**

Both the preventive and curative healthcare provisions accumulate agents’ health stock and stimulate economies’ productivities. However, with limited medical resources, increases in preventive health expenditure crowd out curative expenditure, and vice versa, which in turn impairs the population’s health and deters economic growth. This research aims to provide a empirically rigorous test on the hypothesis that optimally allocating health expenditure between prevention and cures stimulates economic growth within different countries, especially developed countries, and investigates whether health services are luxury goods on the path of economic development.

**Methods:**

Based on OECD country experiences, this present study uses the system generalized method of moments (GMM) estimation method to examine the roles of preventive and curative healthcare services over the path of economic development and proves that preventive and curative health spending have non-linear effects on economic performance.

**Results:**

For growth maximization, the optimal share of preventive health expenditure to GDP is 0.44% with per capita GDP at US$40,465; the real share is 0.25% with per capita GDP at US$35,230. The optimal share of curative health expenditure to GDP is 10.96% with per capita GDP at US$41,816; the real share is 8.26% with per capita GDP at US$35,230. Accordingly, the estimated optimal provision of health services are currently underprovided. This research further estimates the effects of income on demand for care and shows that the income elasticities of preventive and curative health care are greater than unity. Health services are luxury goods.

**Conclusions:**

Economies with higher incomes demand such services more than those with lower incomes. The large positive effects of income on preventive care use exist.

## Introduction

Health expenditure has increased due to economic development, the progress of health technology, and the coming of aging societies in developed countries, and even in developing countries. With longer life expectancy, chronic illness has become the main cause of death and accounts for an estimated 60% of all deaths in the world. In the past, medical care has focused on disease treatment, which could reduce mortality rate and extend life expectancy [1]. There has been a trend towards neglect and a rejection of the hospital, regarding curative services as even counterproductive in the improvement of health stock. Some researchers have supported the position that money spent on curative services could so much better than spent on preventive care [2, 3]. On the other hand, others have concluded that preventing illness can in some cases save money [4, 5]. According to Organization for Economic Co-Operation and Development (OECD) indicators of risk factors to health, the United States and Mexico are facing difficulties of overweight and obesity among children and adults. Countries in Europe, such as Austria and France, are facing the difficulties linked to tobacco and alcohol consumption. These factors possibly lead to chronic illness and raise health expenditure. Early prevention and intervention could lower the probability that the population will be in poor health and save subsequent curative expenditure.

Prevention has been defined and perceived in many different ways, so it is critical to have a concrete definition to make sure readers are on the same page. This study divides health expenditures into preventive and curative expenditures. Based on the main function of health expenditures, the OECD Health Statistics divides health expenditures into expenditures of personal and collective health care. Personal health services are composed of inpatient care, outpatient care, long-term care, and pharmaceuticals. Collective health services are composed of prevention and public health services, administration, and health insurance. Prevention/public health and administration expenditures could be defined as prevention expenditure [6]. Total health expenditure minus preventive expenditure is curative expenditure. Although different countries may include expenditures of preventive spendings with variations, those listed by OECD prevention seem to be largely comparable and policy relevant.

Health services could be regarded as consumption and investment goods [7]. Curative expenditure treats the disease as well as eases the pain, and is thus concerned as consumption spending. Preventive health care accumulates health stock as well as increases the number of days available to participate in market and non-market activities, which in turn stimulates goods production. Preventive expenditure could be investment goods. The preventive health expenditure in developed countries has usually reduced complications and/or mortality, although this could also have ambiguous effects on cost saving and economic performance. How much health spending should be allocated between cure and prevention? The answer to this important question leads to a fundamental question being asked: Can the investment on prevention raise the population’s well-being, which can be inferred by increases in income [8]?

In real-world data, countries with higher incomes often have higher health expenditure than those with lower incomes. Health services are luxury goods and the increase in health spending is bigger than the increase in national income [8, 9]. Most of previous studies have focused on the discussions regarding the relationship between total health expenditure and economic development [10, 11]. Wang et al., among the few, provided an analytical framework with Taiwanese and American experiences to demonstrate the influences of the provision of preventive health services on economic performance and to find an optimal share of preventive health expenditure to gross domestic product (GDP) [6]. Both the preventive and curative healthcare provisions accumulate agents’ health stock and stimulate economies’ productivities. However, with limited medical resources, increases in preventive health expenditure crowd out curative expenditure, and vice versa, which in turn impairs the population’s health and deters economic growth. For maximizing economic growth, health expenditure should be allocated optimally between preventive and curative health services. This research aims to provide a more empirical rigorous test of the hypothesis that allocating health expenditure optimally stimulates economic growth within different countries, especially developed countries, and investigates whether health services are luxury goods on the path of economic development [8, 9].

Based on OECD country experiences, this study offers new evidence regarding (a) prevention-cure-growth nexus, and (b) the income effects on preventive and curative health services demanded. This study contributes to and improves upon earlier works in the following ways: First, health expenditure is decomposed of preventive and curative expenditure, thus permitting us to examine the growth effects of medical resource allocations. Second, this study is among the first to examine how the preventive and curative health services are competitive in medical resource allocations but complementary for improving the population’s well-being, which could be inferred by better economic performance and enhanced health status. Third, we determine what the effects of income are on the demand for health care. This research estimates the elasticities with respect to income and predicts changes in the consumption of preventive and curative health services in response to income variations.

## Methods

The use of system generalized method of moments (GMM) has been widely applied to estimate everything from financial and economic issues to public health studies on the effects of AIDS deaths on households since the 1990s [12, 13]. These econometric techniques are specifically designed to extract causal lessons from the data or observations (whether countries, hospitals, or patients), each of which is observed only annually over five or ten years, as well as to resolve multicollinearity difficulties between explanatory variables. If income were also a function of preventive health services, ordinary least squares (OLS) estimates would be biased and inconsistent. This paper introduces system GMM to drive the design of the estimators of interest with independent variables that are correlated with past and possibly current realizations of the error. This approach gives us confidence in the reported coefficients and standard errors.

For maximizing economic growth, an optimum of healthcare provision does exist [6]. The econometric specification in this study is presented as follows:

$$\begin{array}{c} {Lny}_{it}=\alpha_{it}\phi_{i,nt}{+\beta }_{it}\phi_{i,nt}^{2}+ϒX_{it}{+\eta }_{it}{+\delta }_{t}{+\varepsilon }_{it}\\ t=1,2,\ldots ,T;i=1,2,\ldots ,N. \end{array}$$ (1)

Following standard practice [14, 15], our indicator for economic development is per capita real gross domestic product (GDP). The dependent variable *Lny*_*it*_ represents per capita real GDP in natural logarithm form (based on year 2010 US dollars) because the growth rate of a variable equals the derivative with respect to time of its natural logarithm, ϕ_*i*,*nt*_ represents the fractions of preventive and curative health expenditure over GDP, *n* = 1, 2 present the preventive and curative health expenditure, *η*_*i*_ represents a country-specific effect, δ_*t*_ represents a time-year effect, ε_*it*_ represents an idiosyncratic error term, and *i* and *t* represent country and time period, respectively. Referring to Barro and Sala-i-Martin [16], the variables *X*_*it*_ are composed of the life expectancy for the health indicator to examine the influence of the population’s health status on economic prosperity [17], the ratio of education expenditure to GDP for presenting the quality of human capital [18], the population for presenting the quantity of human capital [19], the ratio of savings to GDP for capturing the accumulation of private capital [20], the ratio of trade to GDP for capturing openness [21], the treasury bill rate for presenting the interest rate and the price of physical capital [22], the ratio of the elderly to the population for examining the influences of aging, and the fraction of the population aged 60+ on economic performance [23]. The influence which each variable has on productivity and growth has been of particular interest in the literature. This research then includes the aforementioned concerns.

### Data

There are 36 OECD member countries existing today. Chile, Estonia, Israel, Latvia, Lithuania, and Slovenia participated in the organization recently with short data series. Ireland might not decompose the total health expenditure into prevention and treatment spending. Applying 29 OECD country experiences, the panel datasets of dependent and explanatory variables were observed over the period from 1998 to 2013. These countries are composed of: Australia, Austria, Belgium, Canada, Czech Republic, Denmark, Finland, France, Germany, Greece, Hungary, Iceland, Italy, Japan, South Korea, Luxembourg, Mexico, the Netherlands, New Zealand, Norway, Poland, Portugal, Slovak Republic, Spain, Sweden, Switzerland, Turkey, the United Kingdom and the United States. The data is collected from OECD statistics, which offers comprehensive sources on health, healthcare systems, and determinants of economic growth for carrying out empirical analyses across OECD countries and deriving lessons for diverse healthcare systems. Table 1 summarizes the descriptive statistics and shows the means, standard deviations (Std. Dev.), the minimum, and the maximum. According to these, this study has relatively large ranges of values since the estimated samples are more comprehensive. In our sample, Luxembourg has the highest per capita real GDP (US$89,911); Poland has the lowest per capita real GDP (US$11,570). In addition, New Zealand and Slovak Republic, respectively, have the highest and the lowest shares of preventive health expenditure to GDP, at 0.7% and 0.001%. The highest and lowest shares of curative expenditure to GDP are in the United States with 15.91% and South Korea with 3.62%, respectively. The longest and shortest life expectancy at birth are at Japan and Turkey with 83.4 and 70.3, respectively.

**Table 1:** Summary statistics.

Variables	Mean	Std. Dev.	Minimum	Maximum
Per capita GDP (US dollar)	35072.25	13485.58	11570.20	89911.10
*Ln* (per capita GDP)	10.39	0.39	9.36	11.41
Life expectancy	78.75	2.75	70.30	83.40
Education expenditure/GDP (%)	5.23	1.22	2.59	8.62
The population (millions)	40.8	59.2	0.27	317
The elderly/the population (%)	14.75	3.57	4.87	25.01
Preventive health expenditure/GDP (%)	0.25	0.14	0.00	0.70
Curative health expenditure/GDP (%)	8.26	1.93	3.62	15.92
Savings/GDP (%)	6.52	6.18	–13.53	27.62
Trade/GDP (%)	84.89	53.22	18.76	357.48
Treasury bill rate (%)	3.951	3.546	0.02	27.14

Source: Author’s calculations from OECD statistics, http://stats.oecd.org/

## Results

This study provides quantitative estimates of the impact of interested concerns on the reported economies’ performances. The system GMM estimated results for economic performance, per capita real GDP in natural logarithmform, to selected variables are presented in Table 2 and convey a wealth of information. On the examination of preventive health spending, a 10% increase in the heath status (the life expectancy at birth) increases per capita real GDP growth rate by 0.81%. The reason is that better health status leads to higher productivities and hence better economic performance. A 10% increase in the ratio of an economy’s savings to GDP increases per capita real GDP growth rate by 0.22%. The explanation is that savings acculumate physical capital and stimulate production. The other socio-economic and demographic explanatory variables, including education, population, aging status, openness, and the interest rate, do not affect economic performance significantly. The statistics of AR(1), autoregressive in first differences in first lag; AR(2), autoregressive in first differences in second lags, Hansen test, and Sargan test are, in sequence, 0.166, 0.714, 0.269, and 0.172, and present that the selection of variables are valid, the estimation equation is correctly specified, and the estimation result is robust.

**Table 2:** System GMM regression estimates of *Ln* (per capita GDP) on selected variables.

Period of test = 1998~2013		Number of observations = 303	Number of groups = 29	
*Ln* (per capita GDP)		Preventive health care provision	Curative health care provision	
Constant		3.526**	4.053***	
		(1.445)	(1.408)	
Prevention heath expenditure/GDP (%)		3.038**	―	
		(1.321)	―	
The square of prevention heath expenditure/GDP (%)		–3.582**	―	
		(1.917)	―	
Curative heath expenditure/GDP (%)		―	0.504***	
		―	(0.176)	
The square of curative heath expenditure/GDP (%)		―	–0.023**	
		―	(0.012)	
Life expectancy		0.081***	0.046*	
		(0.022)	(0.025)	
Education expenditure/GDP (%)		0.019	0.049	
		(0.053)	(0.098)	
The population (millions)		—	—	
		(—)	(—)	
The elderly/the population (%)		–0.000	–0.015	
		(0.023)	(0.028)	
Trade		—*	—	
		(0.002)	(0.002)	
Savings/GDP (%)		0.022***	0.022	
		(0.008)	(0.015)	
Treasury bill rate (%)		0.009	0.016*	
		(0.008)	(0.01)	
AR(1)		0.166	0.914	
AR(2)		0.714	0.978	
Sargan test		0.269	0.636	
Hansen test		0.172	0.124	

Note

***: significant at 1%

**: significant at 5%

*: significant at 10%; the numbers in parentheses are standard errors.

Dashed lines indicate a significance less than one thousandth and a prevalence less than 5 per 1000.

On the examination of curative health spendings, a 10% increase in the health status (the life expectancy at birth) increases per capita real GDP growth rate by 0.46%. A 10% increase in the interest rate (the treasury bill rate) increases per capita real GDP growth rate by 0.16%. The explanation is that a rising interest rate is a strong indicator of economic growth since more businesses reach out to banks and other financial lenders for extensions of capital. The statistics of AR(1), AR(2), Hansen test, and Sargan test are, in sequence, 0.914, 0.978, 0.636, and 0.124, and present that the selection of variables are valid, the estimation equation is correctly specified, and the estimation result is robust.

**Fig 1:**
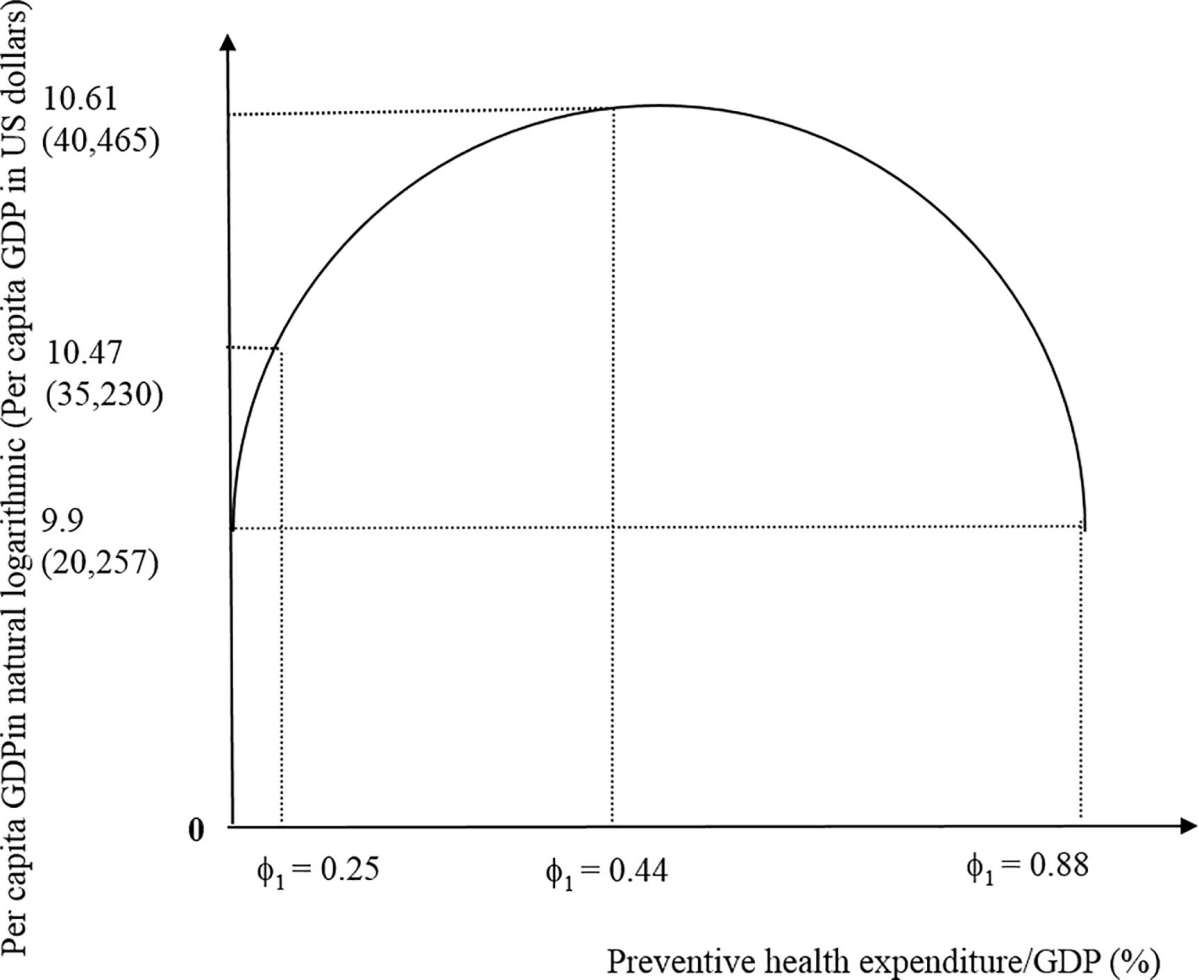
The relationship between preventive health expenditure and economic performance.

**Fig 2:**
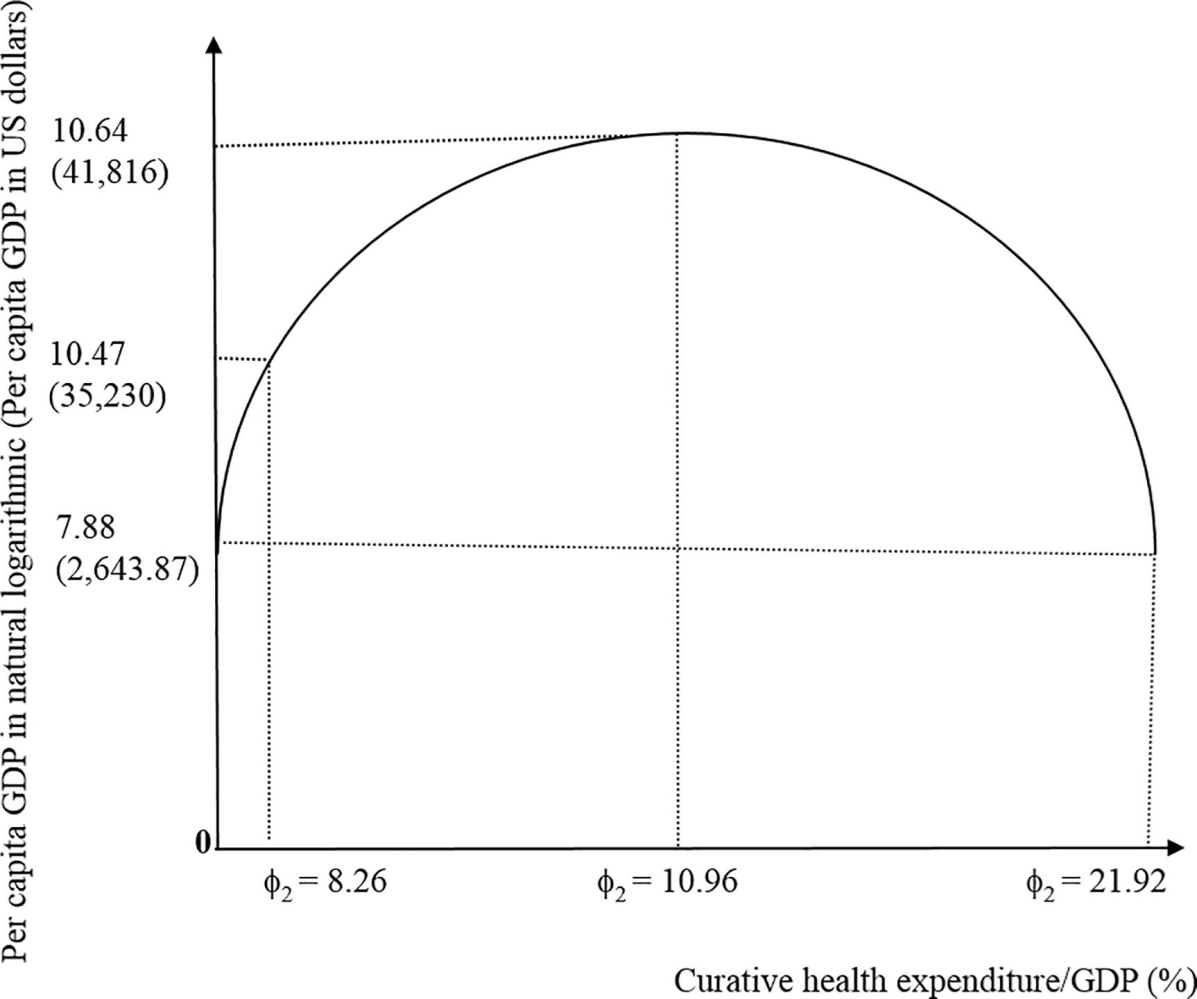
The relationship between curative health expenditure and economic performance.

By ruling out trivial cases, the influences of significant variables are then averagely specified in the constant term. The impact of preventive and curative health services on economic performance, respectively, are estimated as:

$$Ln\;(\mathrm{per}\;\mathrm{capita}\;\mathrm{GDP})=9.9+3.203\phi_{1}-3.618\phi_{1}^{2}$$ (2)

$$Ln\;(\mathrm{per}\;\mathrm{capita}\;\mathrm{GDP})=7.88+0.504\phi_{2}-0.023\phi_{2}^{2}$$ (3)

in which ϕ_*i*, *i* = 1, 2_ presents the shares of preventive and curative health expenditure to GDP. Differentiating Eqs (2) and (3), we find that the optimal share of preventive health expenditure to GDP is 0.44% with per capita GDP in natural logarithm at 10.61 or per capita GDP in level at US$40,465; the real share is 0.25% with per capita GDP in natural logarithm at 10.47 or per capita GDP in level at US$35,230. Fig 1 presents the inverse U-shaped relationship between the provision of preventive health services and economic performance. A non-linear relationship exists between preventive health expenditure and economic growth, which is consistent with previous theoretical findings [6]. With zero preventive health expenditure or the share 0.88%, economies have per capita GDP in natural logarithmic at 9.9 or per capita GDP in level at US$20,257. Though Wang et al. [6] used the OLS (ordinary least square) estimation method and tested the US experiences over the period from 1975 to 2013 to verify the existence of the nonlinear prevention-growth nexus, the applications of exact optimal level of cross countries were not discussed. Raising spendings on prevention improves the population’s health, increases productivities, and stimulates economic growth. However, more preventive expenditure crowds out curative expenditure or other infrastructure expenditure and impedes economic growth. The current research finds that an optimum of preventive health expenditure for maximizing economic growth in OECD countries does exist, but may not have been noticed in earlier works. Fig 2 presents that the optimal share of curative health expenditure to GDP is 10.96% with per capita GDP in natural logarithm at 10.64 or per capita GDP in level at US$41,816; with zero curative health expenditure or the share 21.92%, economies have per capita GDP in natural logarithm at 7.88 or per capita GDP in level at US$2,643.87; the real share is 8.26% with per capita GDP in natural logarithm at 10.47 or per capita GDP in level at US$35,230.

### Income effects on prevention and cures demanded

The share of GDP devoted to preventive (curative) care in OECD countries increased from 0.23% (7.62%) in 1998 to 0.26% (9.08%) in 2013. Per capita GDP has also increased steadily about 46% during this period. Based on 2010 constant dollars, per capita preventive spending has increased around 37% between 1998 and 2013, and per capita curative spending has increased 46% in that period, as Table 3 presents. This brief excursion through 29 OECD countries’ healthcare spending suggests that aggregate per capita spending on health care turns out to be strongly related to per capita income, as showed by Newhouse [8] in the 1970s. The magnitude of the changes in per capita healthcare expenditure as per capita GDP changes.

**Table 3:** Relation of healthcare expenditure and income for OECD countries in 2010 constant US dollars from 1998 to 2013.

Year	1998	2013	Increases (%)
Preventive expenditure/GDP (%)	0.23	0.26	0.03
Curative expenditure/GDP (%)	7.62	9.08	1.46
Per capita GDP	3,0375.24	3,7139.62	46
Per capita preventive expenditure	70.91	97.43	37
Per capita curative expenditure	2,314.44	3,371.41	46

Source: Author’s calculations from the OECD statistics, http://stats.oecd.org/

The natural logarithmic regression with these data shows a big estimated income elasticity of demand for preventive health care:

$$\begin{array}{c} Ln\left(\mathrm{pre}\;\mathrm{capita}\;\mathrm{preventive}\;\mathrm{spending}\right)=-11.54+1.52[Ln\;(\mathrm{per}\;\mathrm{capita}\;\mathrm{GDP})]\\ \left(t/SE=-12.22/0.944)\;(t/\mathrm{SE}=16.75/0.09\right)\\ N=407\;R^{2}=0.41 \end{array}$$ (4)

The abbreviations *t* and *SE* in the parentheses of Eq (4) respectively present *t* statistics and standard error. In natural logarithmic data, the estimated coefficient on income is the income elasticity itself. The income elasticity of prevention is estimated at 1.52. When income increases 1%, the prevention demanded increases by 1.52%. Over 40 percent of the variance in per capita preventive expenditure in OECD countries can be explained by variation in per capita GDP in making international comparisons. The intercept and the coefficient of *Ln* (per capita GDP) are significant at 0.1% level. Simultaneously, the natural logarithmic estimated equation for curative health spending is:

$$\begin{array}{c} Ln\;\left(\mathrm{per}\;\mathrm{capita}\;\mathrm{curative}\;\mathrm{spending}\right)=-6.2+1.35[Ln\;(\mathrm{per}\;\mathrm{capita}\;\mathrm{GDP})]\\ \left(t/SE=50.72/0.278)\;(t/SE=-22.27/0.027\right)\\ N=407\;R^{2}=0.86 \end{array}$$ (5)

The elasticity of curative spending with respect to income is 1.35. When income increases 1%, the prevention demanded increases by 1.35%. The intercept and the coefficient of *Ln* (per capita GDP) are significant at 0.1% level. A strong statistical relationship exists between curative expenditure and income. Eqs (4) and (5) show positive effects of income on the demand for health care. The income elasticities of health services exceed one and the marginal preventive and curative health services are luxury goods [8].

**Fig 3:**
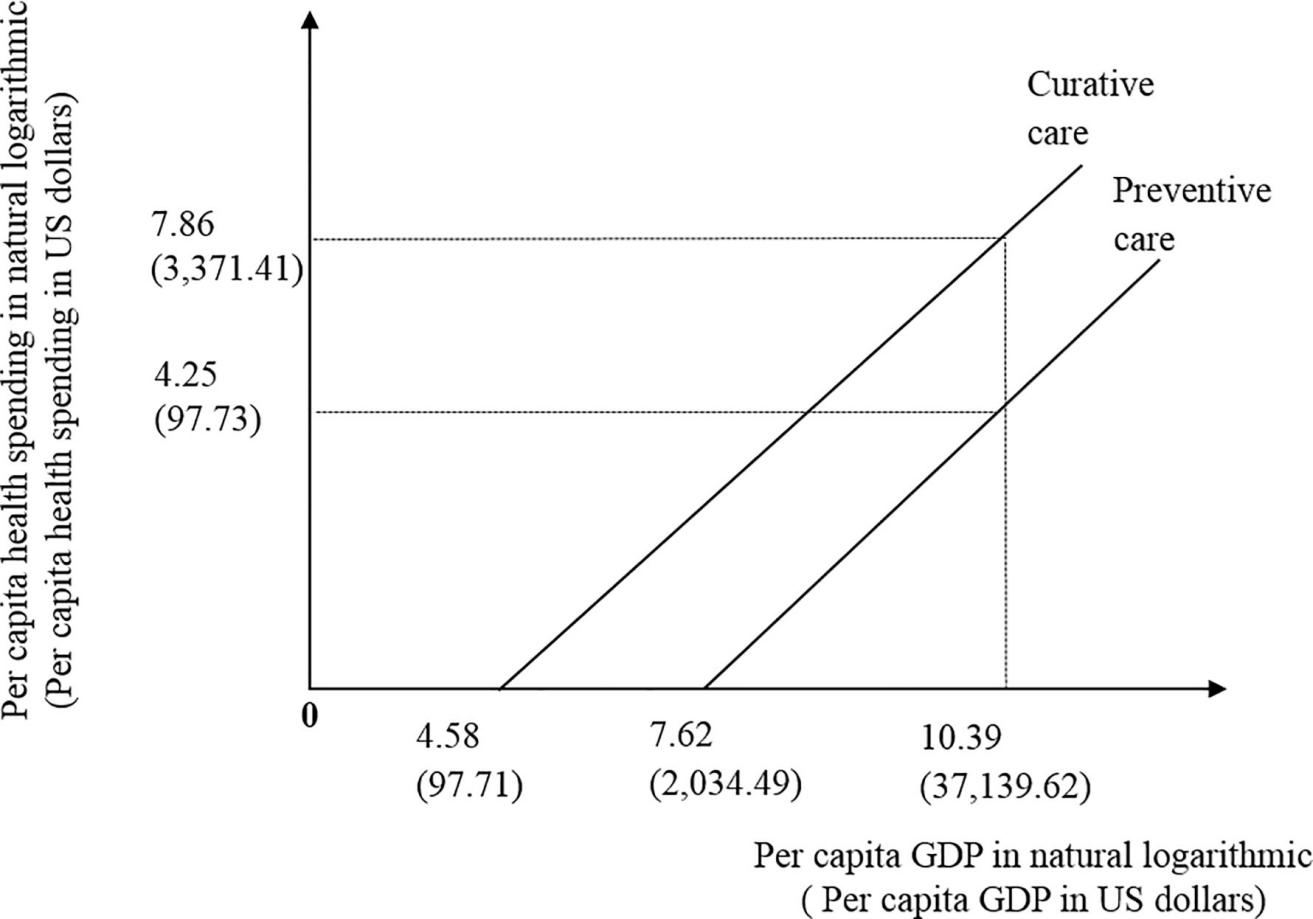
Engel curves for preventive and curative care.

Fig 3 depicts the Engel curves for presenting the interrelationships that begin with increases in per capita GDP, which further induce the demand for preventive and curative healthservices, thereby causing the population’s health to improve. On the relationships between per capita GDP and health services demanded, the society begins to request respectively curative and preventive care, when per capita is greater than 4.58 and 7.62 in natural logarithmic or US$ 97.71 and US$2,034.49. In 2013, per capita GDP is at US$37,139.62 or 10.39 in natural logarithmic with per capita preventive expenditure around at US$97.73 or 4.25 in natural logarithmic and per capita curative expenditure around at US$3,371.41 or 7.86 in natural logarithmic. The needs for both health services increase in income. The large positive effects of income on preventive care use exist.

## Discussions

Over the coming five decades, not only developed countries but also many developing countries will face sharp rises in the health expenditure and similar healthcare challenges. Providing health services to protect against entering a worse health status and to promote better life for chronically ill patients is a recent phenomenon in most of these countries. This study proposes that early prevention and examination could improve health stock and save subsequent curative expenditure, which in turn promotes productivities and stimulates economic growth. We examined the role of preventive and curative healthcare expenditure over the path of economic development and whether OECD countries could stimulate economic performance by appropriately allocating health expenditure. The empirical results reveal that preventive and curative spendings has non-linear effects on economic growth. The non-linear prevention-growth nexus is in line with that found by Wang et al. [6] which provided theoretical analysis and applied the method of ordinary least square (OLS) to estimate such relationship on the U.S. experiences over the period 1975–2013. This research further estimates the effects of income on demand for care and shows that the income elasticities of preventive and curative health expenditure are greater than unity. Preventive and curative health care are luxury goods. Newhouse offered the estimation of the elasticities of per capita medical care with respect to per capita GDP, basing on 13 developed countries in 1972 and reached the similar result that income elasticities substantially exceed one [8]. Economies with higher incomes demand health care more than those with lower incomes.

It is possible that ongoing preventative expenditure followed by later treatment expenditure will prove more costly in the long run [2, 3]. This study indicates that a bigger preventive health expenditure share is associated with better economic performance. However, after a critical prevention share, the negative impacts on the population’s health and the economy’s growth are expected. Figs 1 and 2 illustrate how preventive and curative healthcare provision influence the economic performance. Preventive healthcare provision reduces later treatment expenditure by allowing future population cohorts and healthcare providers to meet healthier life style because of greater awareness of the risk and more widespread detection [4, 5].

With economic development and longer life expectancy, the growth rates of expenditure on outpatient care and long-term care are higher than those on prevention and public health. Though curative health services are important for the improvement of health and are likely to remain so in the future, prevention offers ways for the delivery of health care for maintaining and even accumulating health stock as well as new approaches to retain good economic performance. Economies with few or even without prevention inputs will increase disease prevalence and require more cure expenditures.

## Conclusions

The provision of preventive health care is as important as curative health care for reducing causes of death, disease, and disability. Appropriately investing in prevention and devoting to cures could raise a population’s well-being, which can be inferred by increases in income and health stock [8, 17]. On medical resource allocations, preventive and curative health services are competitive. Nevertheless, both health services are complementary for enhancing health status. This study has presented guidelines to assessment for use in program evaluation, monitoring of health policy, and health services research. The empirical results are applicable to other developed and developing countries for evaluating the effectiveness of preventive and curative health care.
